# Somatic *GJA4* gain-of-function mutation in orbital cavernous venous malformations

**DOI:** 10.1007/s10456-022-09846-5

**Published:** 2022-07-29

**Authors:** Hiroki Hongo, Satoru Miyawaki, Yu Teranishi, Jun Mitsui, Hiroto Katoh, Daisuke Komura, Kinya Tsubota, Takashi Matsukawa, Masakatsu Watanabe, Masakazu Kurita, Jun Yoshimura, Shogo Dofuku, Kenta Ohara, Daiichiro Ishigami, Atsushi Okano, Motoi Kato, Fumihiko Hakuno, Ayaka Takahashi, Akiko Kunita, Hiroyuki Ishiura, Masahiro Shin, Hirofumi Nakatomi, Toshitaka Nagao, Hiroshi Goto, Shin-Ichiro Takahashi, Tetsuo Ushiku, Shumpei Ishikawa, Mutsumi Okazaki, Shinichi Morishita, Shoji Tsuji, Nobuhito Saito

**Affiliations:** 1 Department of Neurosurgery, Faculty of Medicine, The University of Tokyo, 7-3-1 Hongo, Bunkyo-ku, Tokyo, 113-8655 Japan; 2 Department of Molecular Neurology, Graduate School of Medicine, The University of Tokyo, Tokyo, Japan; 3 Department of Preventive Medicine, Graduate School of Medicine, The University of Tokyo, Tokyo, Japan; 4 Department of Ophthalmology, Tokyo Medical University, Tokyo, Japan; 5 Laboratory of Pattern Formation, Graduate School of Frontier Biosciences, Osaka University, Suita, Osaka Japan; 6 Department of Plastic, Reconstructive and Aesthetic Surgery, The University of Tokyo Hospital, Tokyo, Japan; 7 Department of Computational Biology and Medical Sciences, Graduate School of Frontier Sciences, The University of Tokyo, Kashiwa, Chiba Japan; 8 Department of Animal Resource Sciences, Graduate School of Agriculture and Life Sciences, The University of Tokyo, Tokyo, Japan; 9 Department of Pathology, Graduate School of Medicine, The University of Tokyo, Tokyo, Japan; 10 Department of Neurology, Faculty of Medicine, The University of Tokyo, Tokyo, Japan; 11 Department of Anatomic Pathology, Tokyo Medical University, Tokyo, Japan; 12 Institute of Medical Genomics, International University of Health and Welfare, Narita, Chiba Japan

**Keywords:** Vascular malformations, Orbital disease, Connexin, Gap junction protein, Whole-cell voltage clamp, Endothelial cell

## Abstract

Orbital cavernous venous malformation (OCVM) is a sporadic vascular anomaly of uncertain etiology characterized by abnormally dilated vascular channels. Here, we identify a somatic missense mutation, c.121G > T (p.Gly41Cys) in *GJA4*, which encodes a transmembrane protein that is a component of gap junctions and hemichannels in the vascular system, in OCVM tissues from 25/26 (96.2%) individuals with OCVM. GJA4 expression was detected in OCVM tissue including endothelial cells and the stroma, through immunohistochemistry. Within OCVM tissue, the mutation allele frequency was higher in endothelial cell-enriched fractions obtained using magnetic-activated cell sorting. Whole-cell voltage clamp analysis in *Xenopus* oocytes revealed that *GJA4* c.121G > T (p.Gly41Cys) is a gain-of-function mutation that leads to the formation of a hyperactive hemichannel. Overexpression of the mutant protein in human umbilical vein endothelial cells led to a loss of cellular integrity, which was rescued by carbenoxolone, a non-specific gap junction/hemichannel inhibitor. Our data suggest that *GJA4* c.121G > T (p.Gly41Cys) is a potential driver gene mutation for OCVM. We propose that hyperactive hemichannel plays a role in the development of this vascular phenotype.

**Supplementary Information:**

The online version contains supplementary material available at 10.1007/s10456-022-09846-5.

## Introduction

Orbital cavernous venous malformation (OCVM), one of the most common benign orbital lesions in adults [1], occurs more often in women and typically presents in the fourth and fifth decades of life [2]. OCVM grows slowly over time and typically cause symptoms, including progressive exophthalmos, visual deterioration, and strabismus by mass effect [2]. Histologically, OCVM is characterized by dilated vascular channels lined by mature, flattened endothelial cells surrounded by abundant fibrous stroma [3]. Smooth muscle cells and elastic fibers are sparsely found in the stroma, and the vascular cavity is filled with thrombosis, reflecting the stasis of blood flow [2]. Symptomatic lesions require surgical intervention, the approach of which is selected depending on the location of the lesions and the surrounding structure, including extraocular muscles, motor and sensory nerves, and the optic nerve [2, 4]. The anatomical complexity of the orbit hinders surgical excision [4]. As a minimally invasive treatment, intralesional injection of pingyangmycin is a treatment option for lesions that are particularly difficult to resect due to their deep location, such as lesions in the orbital apex [5]. Despite advances in managing these lesions, relatively little research has been dedicated to the understanding of their molecular pathophysiology.

Vascular malformations arise due to defects during early vascular development, which lead to locally, abnormally formed vessels [6], involve the entire body, and cause significant morbidity in the affected organs and tissues, requiring multidisciplinary specialists and complex treatment [7]. As a result of a better understanding of their biological behavior, the International society for the study of vascular anomalies (ISSVA) classification was established [8]. ISSVA classifies vascular malformations by their clinical and histologic characteristics into capillary, lymphatic, venous, arteriovenous, and combined malformations, achieving uniformly classifying a wide, heterogeneous spectrum of lesions. There also have been several studies on their genetic causes reporting genetic mutations as a cause [6]. Recent advances in sequencing technology have enabled the identification of somatic mutations in low allele frequency in sporadic lesions. Most mutations have been detected in genes that play important roles in pathways involved in angiogenesis, vascular cell growth, apoptosis, and proliferation, such as RAS/mitogen-activated protein kinase (MAPK)/extracellular signal-regulated kinase (ERK) or the phosphatidylinositol 3-kinase (PI3K)/protein kinase B (AKT)/mammalian target of rapamycin (mTOR) pathway [9]. These discoveries of genetic mutations in patients have allowed for the development of molecular targeted therapies for vascular malformations.

*GJA4* (also known as *Cx37*, MIM: 121012) is a gene in connexin family. Full-length connexins oligomerize and form channel structures connecting the cytosol of adjacent cells (gap junctions) or the cytosol with the extracellular space (hemichannels), which allow for the transfer of ions and small molecules, and mediate cellular communications [10]. Of the 21 different human connexin isoforms, each with its own distinct biophysical properties and expression pattern, GJA4 is one of connexins expressed in the vascular system [11]. In vascular cells, connexin channels play essential roles ranging from electrical coupling, vascular remodeling, angiogenesis, and vascular permeability [11]. Due to their fundamental roles in vascular cell biology, dysfunction of connexin channels in the vascular system is often associated with vascular pathologies [12]. In patients with atrial fibrillation (MIM: 614049), some mutations in *GJA5* (MIM: 121013) have been identified [13]. Those mutations have been linked to the pathology by abnormal gap junction formation or absent of transjunctional electrical coupling. In *GJA4*, one polymorphism (c.1019C > T [p.Pro319Ser]) has been identified as an independent risk factor in the development of atherosclerosis, coronary heart disease, and ischemic stroke [14]. The polymorphism may change the behavior of connexin hemichannel properties in monocytes [15]. In vascular malformations, vascular disorders in which impaired remodeling of vessel networks and angiogenesis can contribute to their pathologies, however, how connexins are involved in their pathology remains unclear.

In this study, using targeted deep sequencing and droplet digital PCR (ddPCR), we identified a recurrent *GJA4* mutation (c.121G > T [p.Gly41Cys]) in OCVMs, whose genetic cause has been previously undefined despite their typical histological characteristics as venous malformations, the most frequent form of vascular malformations. GJA4 expression was detected in OCVM tissue including endothelial cells and the stroma, and within OCVM tissue, the mutation allele frequency was higher in endothelial cell-enriched fractions obtained using magnetic-activated cell sorting. Whole-cell voltage clamp analysis in Xenopus oocytes revealed that *GJA4* c.121G > T (p.Gly41Cys) is a gain-of-function mutation that increases hemichannel activity. In endothelial cells, this mutation disrupted the cell integrity and function, which were rescued using carbenoxolone (CBX), an inhibitor of connexin channels. Our study thus proposes a potential driver gene mutation for a vascular malformation and implicates hyperactive hemichannel as a potential molecular pathogenesis of the vascular phenotype.

## Methods

### Sample preparation

For the discovery cohort, frozen OCVM, cerebral cavernous malformation (CCM), and vertebral hemangioma (VH) samples and paired peripheral whole blood samples were obtained at the Department of Neurosurgery, The University of Tokyo (UT). For the validation cohort, OCVM samples, including both frozen and formalin-fixed paraffin-embedded samples from UT, which were independent of the discovery cohort, and the Department of Ophthalmology, Tokyo Medical University (TMU), were obtained. For the prospective study and magnetic-activated cell sorting, freshly resected tissue samples of two OCVM and one conjunctival capillary hemangioma were obtained at TMU. Commercially available DNA extraction kits (QIAamp DNA Micro Kit and QIAamp DNA formalin-fixed paraffin-embedded (FFPE) Tissue Kit, QIAGEN, Venlo, The Netherlands) were used to isolate genomic DNA from the tissues, according to the manufacture’s protocol. Genomic DNA from peripheral blood leukocytes was isolated at SRL Inc. using the DNA Extraction Kit (Talent, Italy). The study protocol was approved by the Human Genome, Gene Analysis Research Ethics Committee at UT (approval number, G10028) and the Ethics Committee at TMU (approval number, T2020-0051). This study was carried out in accordance with the Declaration of Helsinki of the World Medical Association and the principles set out in the Department of Health and Human Service Belmont Report.

### Targeted deep sequencing

Targeted deep sequencing was performed using TruSeq V3 kits (Illumina, San Diego, CA). For library preparation, the Agilent SureSelect XT Focused Exome target enrichment kit (Agilent, Santa Clara, CA) was used with DNA from tissue or blood samples. Libraries were sequenced on an Illumina HiSeq2500 instrument with 100 bp paired-end sequencing, with a median coverage of 210 ± 82 × for vascular anomalies and 194 ± 61 × for blood samples. Sequencing reads were mapped to the reference genome (hg19) with the Burrows–Wheeler Aligner and then processed with GATK best practice for Somatic SNVs + Indels (default setting of FilterMutectCalls). The datasets supporting this study were submitted to Japanese Genotype–phenotype Archive (JGA) (accession number JGAS000325).

### Sanger sequencing

Bidirectional Sanger DNA sequencing assays were performed to confirm candidate mutations. The sequence of the used primers are as follows: forward primer (CCGTGGTGGGTAAGATCTGG), reverse primer (GCCTGGTCATAGCAGACGTT). PCR was performed using the Veriti 200 thermal cycler (Applied Biosystems, Waltham, MA). KOD DNA Polymerase (TOYOBO Co., Ltd., Osaka, Japan) was used in accordance with the manufacturer’s instructions. Cycle sequencing was performed at FASMAC (Atsugi, Japan) with the ABI Genetic Analyzer 3130XL or ABI DNA Analyzer 3730xL (Applied Biosystems).

### ddPCR

Detection of *GJA4* c.121G > T (p.Gly41Cys) was performed on the QX200 Droplet Digital PCR system (Bio-Rad Laboratories, Inc., Hercules, CA) at UT. Data was analyzed using QuantaSoft v1.4 (Bio-Rad Laboratories). The sequence of the used primers and probes for ddPCR are as follows: forward primer (TTCCGCATCCTCATC), reverse primer (AGGCCTGGTCATAG), fluorescent wild-type probe (5′-HEX-CCTGGCCTGCGAGTC) and mutation allele probe (5′-FAM-CCTGGCCGGCGAGTC). The 20 μl reaction mix consisted of 10 μl of ddPCR Supermix for Probes (no dUTP) (Bio-Rad Laboratories), 1 μl of Custom ddPCR FAM assay (catalog. no.10031276, Bio-Rad Laboratories), 1 μl of Custom ddPCR HEX Assay (catalog. no.10031279, Bio-Rad Laboratories), genomic DNA (40 ng for magnetic-activated cell sorting [MACS]-sorted cells, frozen tissues, and blood samples, or 40 ng or more for FFPE tissue to obtain sufficient copies of the target from fragmented DNA), and DNase/RNase free water up to a total 20 μl. Cycling conditions for the reaction were 95 °C for 10 min, followed by 40 cycles of 94 °C for 30 s and 56 °C for 1 min, then 98 °C for 10 min, and finally a 4 °C hold on a Bio-Rad S1000 Thermal Cycler. As positive control DNAs for each assay, we used pcDNA6/myc-His A *GJA4* wild-type (WT) and—c.121G > T (p.Gly41Cys) plasmids. In addition, distilled water was genotyped with the study samples as non-template controls. ddPCR results of samples that did not have ≥ 500 droplets for *GJA4* WT were considered unreliable and excluded. For larger OCVM samples, DNA isolation and ddPCR were performed for two or three replicates. The results of frozen tissue DNA or FFPE tissue DNA with the highest number of *GJA4* WT droplets in the absence of frozen tissue were selected as definitive mutation allele frequency (MAF). We set a minimum of 0.5% fractional abundance to call a sample positive (similar to [16]).

### Magnetic-activated cell sorting (MACS)

Surgically resected fresh tissues of two OCVMs and one conjunctival capillary hemangioma were cut into pieces and incubated with Collagenase/Hyaluronidase (STEMCELL technologies, Vancouver, Canada) for 30 min at 37 °C with regular agitation; then, vascular endothelial cells were isolated using the CD31 MicroBead Kit, human (Miltenyi Biotec, Bergisch Gladbach, Germany) according to the manufacturer’s recommendations. From CD31-positive and CD31-negative cell fractions, we extracted genomic DNA using the QIAamp DNA Micro Kit.

### Immunohistochemistry

FFPE samples were embedded in paraffin after fixation with 10% formaldehyde. 4 μm slices were cut from FFPE samples, mount on coated slides, and dried for 3 h at 42 °C. After deparaffinization, rehydration, and wash, antigen activation was performed by heating in boiled citrate buffer solution (pH 6.0). Samples were incubated for 1 h at 20 °C in a 4% Donkey Serum/1% BSA/Tris-buffered saline with Tween 20 (TBS-T) solution followed by 4 °C overnight incubation with primary antibodies in 2% Donkey Serum/TBS-T. After five times washes with TBS-T, samples were exposed to secondary antibodies for 1 h at 20 °C. For GJA4 staining, samples were incubated for 1 h in biotinylated antibody, followed by incubation in streptavidin secondary antibody in 2% Donkey serum/TBS-T for 1 h at 20 °C. Three more washes with TBS-T were followed by incubation with the TOTO®-3 nuclear stain. Images were recorded on the ZEISS Axio Imager M1 (Carl Zeiss, Oberkochen, Germany). Following antibodies were used: GJA4 (Abcam ab181701, 1:500), CD31 (R&D Systems AF3628, 1:200), αSMA (Abcam ab21027, 1:200), Ki-67 (Dako, M7240, 1:200), Donkey anti-Rabbit IgG, biotin-SP (Merck Millipore AP182B, 1:500), Streptavidin, Alexa Fluor 488 (Thermo Fisher Scientific S11223, 1:200), Donkey anti-Goat IgG, Alexa Fluor 546 (Invitrogen A-11056, 1:200), TOTO®-3 (Thermo Fisher Scientific T3604, 1:500).

### Preparation of connexin complementary RNA (cRNA) and injection into xenopus oocytes

Whole-cell voltage clamp recording with *Xenopus* oocytes were performed as described [17]. The experiments were approved by the Animal Experiments Committee and Gene Modification Experiments Safety Committee at Osaka University (approval numbers FBS-14-002-1 and 04294). Complementary DNAs (cDNAs) encoding *GJA4* WT and *GJA4* c.121G > T (p.Gly41Cys) were amplified using PCR and cloned into pGEM-HeFx plasmids. The plasmids were linearized using restriction enzymes and then used as a template for in vitro synthesis of cRNA (mMESSAGE mMACHINE T7 Transcription Kit, Invitrogen, Waltham, MA) according to the manufacturer’s protocol. Oocytes were collected from *Xenopus laevis*. An adult *Xenopus* female was anesthetized with ethyl-3-aminobenzoate methanesulfonate, and the ovarian lobes were collected using surgical knife and forceps. The eggs were treated with collagenase solution (20 mg/ml collagenase I (Sigma-Aldrich, Burlington, VT) and 20 mg/ml hyaluronidase (Sigma-Aldrich) in OR2 buffer (82.5 mM NaCl, 2 mM KCl, 1 mM MgCl_2_, and 5 mM HEPES [pH 7.5, adjusted with NaOH]) at 18 °C for 2 h. Stage V and VI oocytes were collected manually and used for cRNA injection. Then 1 ng of *GJA4* WT or *GJA4*- c.121G > T (p.Gly41Cys) cRNA was injected with 10 ng of antisense oligonucleotide DNA for *Xenopus cx38* into *Xenopus* oocytes. Water was co-injected with the antisense oligonucleotide as a negative control. Oocytes injected with cRNA were incubated at 18 °C overnight in ND96 buffer (93.5 mM NaCl, 2 mM KCl, 1.8 mM CaCl_2_, 2 mM MgCl_2_, and 5 mM HEPES; adjusted to pH 7.5 using NaOH). Then, single oocytes were used for hemichannel current recording. For recording transjunctional currents, the vitelline membrane was removed manually using forceps in a hypertonic solution (200 mM aspartic acid, 1 mM MgCl_2_, 10 mM EGTA, 20 mM KCl, and 10 mM HEPES [pH 7.5]) and the oocytes were manually paired with the vegetal poles together prior to incubation.

### Hemichannel current recording

Hemichannel current was measured using the whole-cell voltage clamp technique. Current and voltage electrodes were prepared with a micropipette puller P-1000 (Sutter Instrument, Novato, CA) to obtain a resistance of 0.5–1.0 MΩ. The pipette was filled with solution containing 3 M KCl, 10 mM EGTA, and 10 mM HEPES (pH 7.4). Voltage clamp experiments were performed using the iTEV90 multielectrode clamp amplifier (HEKA, Reutlingen, Germany). To obtain the hemichannel current, the cells were initially clamped at − 40 mV and then subjected to 2 s voltage steps from − 30 to + 50 mV in 10 mV increments.

### Transjunctional (gap junction) current recording

Transjunctional currents were measured using the dual whole-cell voltage clamp technique with two iTEV90 multielectrode clamp amplifiers. To measure transjunctional current, both cells were initially clamped at − 40 mV. One cell was then subjected to 3 s voltage steps from − 140 to + 60 mV in 10 mV increments. Currents detected in the second oocyte were recorded, and junctional conductance was calculated using the current value at the end of the steady state. Conductance was obtained using the equation, G_j_ = I_j_/(V_1_-V_0_), where I_j_ is the current value of the second oocyte. V_0_ and V_1_ are the voltages of the first and second oocyte, whose currents were obtained as I_j_ at each voltage step, V_j_ = V_1−_V_0_. The obtained G_j_ value was then normalized and plotted against the V_j_ values.

### Cell culture

Human umbilical vein endothelial cells (HUVECs) were purchased from Takara Bio Inc. (Kusatsu, Japan). The cells were cultured in EGM-2 medium (Lonza, Basel, Switzerland) supplemented with EGM-2 BulletKit (Lonza), 100 U/ml penicillin, 100 μg/ml streptomycin (FUJIFILM Wako Pure Chemical Corporation, Osaka, Japan), and 10 μM Rho-kinase inhibitor Y27632 (Selleck Chemicals, Houston, TX), which promotes the proliferation of endothelial cells [18].

### Retrovirus production

The experiments were approved by the Committee on Genetically Modified Organisms at UT (approval number 37-5). Full-length *GJA4* WT, *GJA4* c.121G > T (p.Gly41Cys), *GJA4* WT-FLAG (Asp-Tyr-Lys-Asp-Asp-Asp-Asp-Lys), or *GJA4* c.121G > T (p.Gly41Cys)-FLAG were cloned into pMXs vector. GFPNLS was also cloned into pMXs vector for vector control. pMXs vectors were then co-transfected with packaging plasmids (pCMV-gag-pol-PA and pCMV-VSVg) into 293AAV cells (Invitrogen) using Lipofectamine 2000 (Invitrogen). Retroviral supernatants were collected 48 h after transfection.

### Retroviral transduction

For retroviral transduction, HUVECs were seeded at 8,000 cells/cm^2^. The next day, retroviral supernatant of human *GJA4* WT, *GJA4* c.121G > T (p.Gly41Cys), *GJA4* WT-FLAG, *GJA4* c.121G > T (p.Gly41Cys)-FLAG or human GFPNLS (vector control) was obtained and mixed with complete EGM-2 medium in the presence of 4 μg/ml polybrene (Nacalai Tesque Inc., Kyoto, Japan). The medium was changed on days 1, 2, and 4.

### RNA extraction, reverse transcription, and quantitative PCR (qPCR)

Total RNA was extracted with the Quick-RNA Miniprep Plus Kit (ZYMO RESEARCH, Irvine, CA) and reverse transcribed with PrimeScript RT Master Mix (Takara Bio). qPCR was performed using the THUNDERBIRD SYBR qPCR Mix (TOYOBO) and the StepOnePlus Real-Time PCR System (Applied Biosystems). The sequence of the primers used are as follows: forward primer (CCGTGGTGGGTAAGATCTGG), reverse primer (GCCTGGTCATAGCAGACGTT).

### Cell biology experiments

For the assessment of cell shape, cells were observed and imaged on day 5. For the assessment of cellular viability, the MTT (3-[4,5-dimethylthiazol-2-yl]-2,5 diphenyl tetrazolium bromide) assay was performed with the MTT Cell Proliferation Assay Kit (Cayman Chemical), according to the manufacturer’s instructions using Synergy LX (BioTek Instruments, Inc, Winooski, VT) on day 4. For assessing the ability to form a normal capillary network, we performed the tube formation assay. On day 4, 96-well culture plates were coated with 50 μl/well Matrigel (Corning) and incubated for 30 min at 37 °C. After harvesting HUVECs cultured on 6-well plates, 20,000 cells were seeded on coated plates in EGM-2 medium and cultured in a CO_2_ incubator. Thirty minutes before the end of the incubation period, cells were treated with 2 μg/ml calcein AM (FUJIFILM Wako Pure Chemical Corporation) and incubated at 37 °C, 5% (v/v) CO_2_. Tube formation observed at the 6 h time point was imaged. The degree of tube formation was assessed by measuring total mesh area and the number of master junctions using ImageJ (NIH). For experiments assessing the ability of CBX to rescue the phenotypes of HUVECs overexpressing GJA4 p.Gly41Cys, HUVECs were treated with 20 μM CBX (in EGM-2 media) from day 1 to the start of each assay (the start of the MTT assay and the time of seeding on Matrigel for the tube formation assay). Images were taken on an Olympus IX73 inverted fluorescence and bright field microscope using the cellSens software.

### Immunocytochemistry

For immunocytochemistry, cells were fixed using 4% paraformaldehyde, permeabilized using 0.5% Triton-X100, and blocked using 5% normal goat serum. Then, the cells were incubated with the FLAG antibody (FUJIFILM Wako Pure Chemical Corporation 014–22383 1:500) overnight at 4 °C, washed three times, and incubated with goat anti-mouse IgG, Alexa Fluor Plus 555 secondary antibody (Invitrogen A32727, 1:200), and DAPI (Dojindo D523, Kumamoto, Japan, 1:1,000) for 1 h at 20 °C. After further washing, Phosphate-buffered saline (PBS) was added to cells, and images were taken on the Olympus IX73 inverted fluorescence and bright field microscope using the cellSens software.

### Statistical analyses

Comparisons between two groups were performed with a two-tailed t test. *P* value of < 0.05 was considered significant. Analyses were performed using JMP Pro version 15.0.0 (SAS Institute, Inc., Cary, NC).

## Results

### A recurrent somatic *GJA4* mutation in OCVMs

**Fig. 1:**
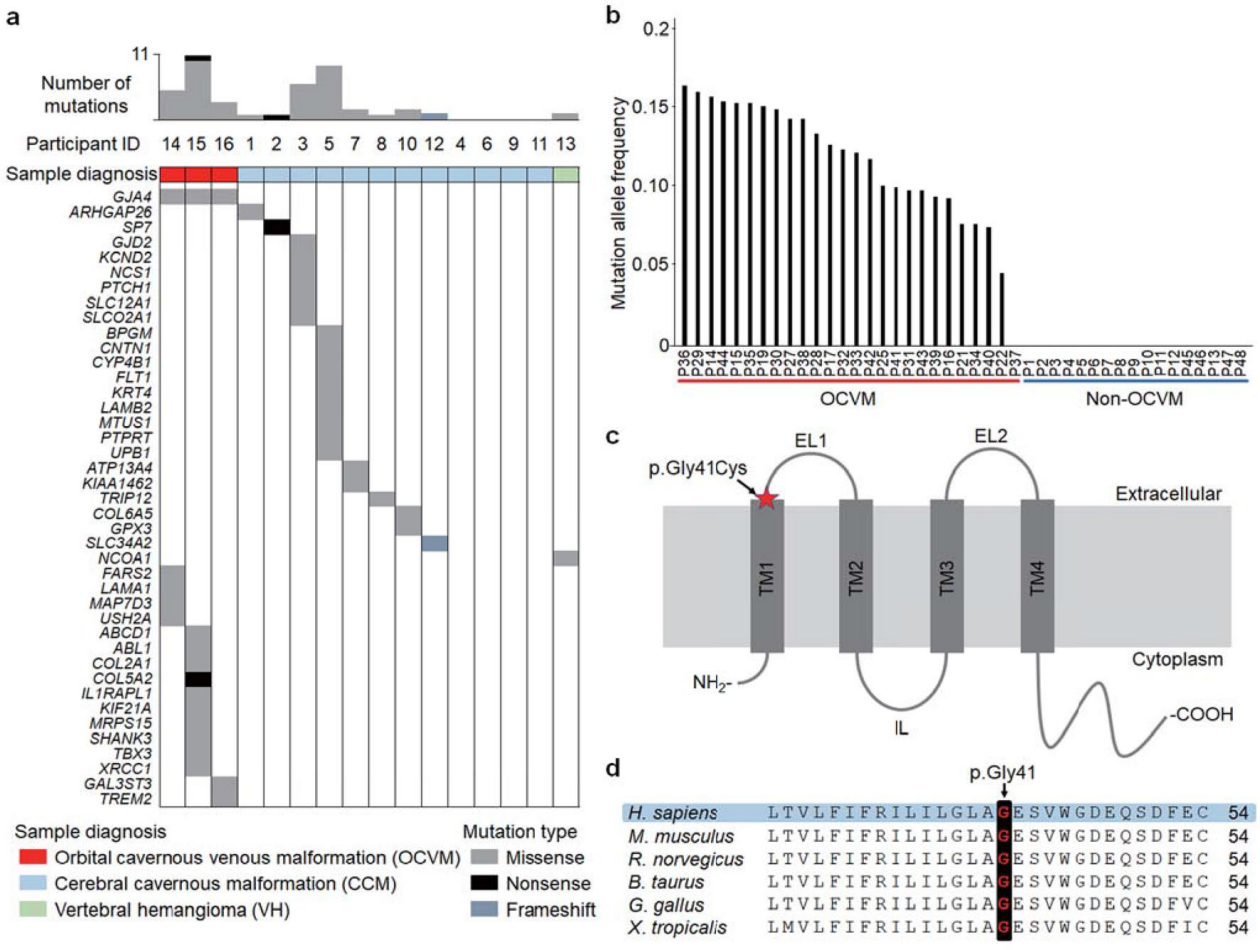
Identification of *GJA4* c.121G > T (p.Gly41Cys) in OCVM tissues. **a** Mutational profiles of vascular anomalies in the discovery cohort. Gene names on the left belong to genes in which somatic mutations were detected. Colored cells represent somatically mutated genes, and color denotes mutation type. **b** MAF of *GJA4* c.121G > T (p.Gly41Cys) was determined using ddPCR analysis in tissue samples from the discovery and validation cohorts. **c** Position of *GJA4* c.121G > T (p.Gly41Cys) in GJA4 (NP_002051.2). TM, transmembrane; EL extracellular loop, IL intracellular loop. **d** Conservation of Gly41 position in GJA4 orthologs. *H. sapiens*, human (NP_002051.2); *M. musculus*, mouse (NP_032146.1); *R. norvegicus*, rat (NP_067686.1); *B. taurus*, cattle (NP_001077207.1); *G. gallus*, chicken (NP_996867.3); *X. tropicalis*, western clawed frog (NP_001072904.1). Sequence alignments, CLUSTALW

To explore somatic mutations in vascular anomalies including OCVM, we performed targeted deep sequencing on 16 clinical samples obtained from participants with vascular anomalies from UT as follows: 3 with OCVM, 12 with CCM (MIM: 116860), and 1 with VH (Table S1 in the Data Supplement). All procedures performed using participant samples were approved by the Human Genome, Gene Analysis Research Ethics Committee at UT (approval number, G10028), and informed consent was obtained from all participants. We extracted genomic DNA from frozen tissue samples of vascular anomalies and paired blood samples. Targeted deep sequencing using SureSelect Focused Exome (Agilent), which is a panel of approximately 6000 genes implicated in human disease, was conducted using the Illumina HiSeq platform. Sequencing coverage of tissues samples of vascular anomalies and paired blood samples was 210 × and 194 × on average, respectively. We identified non-synonymous somatic mutations in all 3 samples with OCVM, 7/12 with CCM, and 1 with VH, with the median number of somatic mutations being 5 (range 3–11), 1 (range 0–9), and 1, respectively (Fig. 1a). Notably, a recurrent somatic *GJA4* mutation was observed in all 3 OCVM samples. This mutation was an identical, missense mutation, *GJA4* (also known as *Cx37*) c.121G > T (p.Gly41Cys) (NM_002060.3, NP_002051.2) (Supplemental Fig. S1), with MAFs of 16.8%, 16.8%, and 8.8%, respectively. Sanger sequencing revealed that for all 3 OCVM tissue samples, the low MAF of *GJA4* c.121G > T (p.Gly41Cys) was shown as signal intensities < 50% (Supplemental Fig. S2). *GJA4* c.121G > T (p.Gly41Cys) is not present in gnomAD and dbSNP (build 138) databases. *GJA4* c.121G > T (p.Gly41Cys) is predicted to be deleterious using in silico prediction models: “damaging” in SIFT, “probably damaging” in Polyphen-2, “disease causing” in MutationTaster, and a score of 24, which indicates that the substitution is among the top 1% of most deleterious ones possible in the human genome using the combined annotation dependent depletion (CADD) algorithm. The mutation was not detected in CCM and VH. Overall, no somatic mutations in genes implicated in vascular anomalies such as *TEK* (MIM: 600221), *PIK3CA* (MIM: 171834), *KRIT1* (MIM: 604214), *CCM2* (MIM: 607929), *PDCD10* (MIM: 609118), *ENG* (MIM: 131195), *ACVRL1* (MIM: 601284), *SMAD4* (MIM: 600,993), *MAP2K1* (MIM: 176872), *RASA1* (MIM: 139150), *KRAS* (MIM: 190070), *BRAF* (MIM: 164757), and *GNAQ* (MIM: 600998) [16, 19–25] were found.

To confirm the presence of *GJA4* c.121G > T (p.Gly41Cys) in the 3 OCVM tissue samples, and validate the mutation in additional OCVM samples, we performed ddPCR using the 3 OCVM tissue samples and paired blood samples in the discovery cohort and independent validation cohort, which consisted of 4 OCVM samples from UT and 24 OCVM samples from TMU (in total 28 OCVM samples). All procedures performed using samples obtained from participants were approved by the Ethics Committee at TMU (approval number, T2020-0051), and informed consent was obtained from the participants. ddPCR of *GJA4* c.121G > T (p.Gly41Cys) was performed using tissue and paired blood samples from 12 participants with CCM and 1 participant with VH in the discovery cohort. In addition, 2 CCM tissue samples (from UT) and 1 tissue sample diagnosed as dilated vein with organizing thrombus (from TMU) were analyzed (Table S2 in the Data Supplement). ddPCR results revealed that all 3 OCVM samples in the discovery cohort had *GJA4* c.121G > T (p.Gly41Cys), with MAFs of 15.7%, 15.6%, and 10.1%, respectively. These MAFs were close to those obtained through targeted deep sequencing. Moreover, the paired blood samples tested negative for the mutation. Of 23 OCVM tissue samples in the validation cohort (2 OCVMs from UT, and 3 OCVMs from TMU were excluded due to the small number of (WT) droplets), 22 (95.7%) contained *GJA4* c.121G > T (p.Gly41Cys), with a median MAF of 12.4% (range 4.6–16.4%). In total, we identified *GJA4* c.121G > T (p.Gly41Cys) in 25 of 26 (96.2%) OCVM tissue samples (Fig. 1b and Table 1). By contrast, *GJA4* c.121G > T (p.Gly41Cys) was not found in any sample with CCM, VH, and dilated vein with organizing thrombus.

**Table 1:** Detection of *GJA4* c.121G > T (p.Gly41Cys) in participants with OCVM

Participant ID	Cohort	Age (years)	Sex	Side	Location	Size (mm)	DNA source	*GJA4* mutation*	Targeted deep sequencing	ddPCR
P14	UT	60	F	L	Intraconal	26	Frozen tissue	c.121G > T	16.8% (75/372)	15.7% (591/3,471)
							Blood	Negative	0% (0/282)	0% (0/4,260)
P15	UT	50	F	L	Intraconal	32	Frozen tissue	c.121G > T	16.8% (54/268)	15.3% (975/5,543)
							Blood	Negative	0% (0/278)	0% (0/3,832)
P16	UT	41	F	L	Intraconal	14	Frozen tissue	c.121G > T	8.7% (35/367)	9.4% (1,152/8,648)
							Blood	Negative	0.4% (1/225)	0% (0/5,426)
P17	UT	36	F	R	Intraconal	7	Frozen tissue	c.121G > T	–	12.7% (611/4,288)
							Blood	Negative	–	0% (0/6,077)
P18	UT	33	M	L	NA	NA	FFPE tissue	NA	–	–
P19	UT	34	F	R	Intraconal	23	FFPE tissue	c.121G > T	–	15.1% (263/1,670)
P20	UT	48	M	L	Intraconal	13	FFPE tissue	NA	–	–
							Blood	Negative	–	0% (0/8,705)
P21	TMU	57	F	R	Intraconal	16	Frozen tissue	c.121G > T	–	4.6% (248/4,695)
P22	TMU	39	F	R	Intraconal	25	Frozen tissue	c.121G > T	–	7.7% (525/5,791)
P23	TMU	58	F	R	Intraconal	15	FFPE tissue	NA	–	–
P24	TMU	66	M	L	Intraconal	13	FFPE tissue	NA	–	–
P25	TMU	63	F	L	Intraconal	17	FFPE tissue	c.121G > T	–	10.1% (484/4,317)
P26	TMU	63	M	L	Intraconal	30	FFPE tissue	NA	–	–
P27	TMU	37	F	L	Intraconal	21	FFPE tissue	c.121G > T	–	14.3% (282/1,891)
P28	TMU	47	M	R	Intraconal	32	FFPE tissue	c.121G > T	–	13.4% (208/1,505)
P29	TMU	34	F	R	Intraconal	28	FFPE tissue	c.121G > T	–	16.0% (267/1,615)
P30	TMU	66	F	R	Extraconal	10	FFPE tissue	c.121G > T	–	14.9% (493/3,078)
P31	TMU	64	F	R	Extraconal	17	FFPE tissue	c.121G > T	–	9.8% (180/1,737)
P32	TMU	48	F	R	Extraconal	12	FFPE tissue	c.121G > T	–	12.4% (160/1,253)
P33	TMU	39	F	L	Extraconal	27	FFPE tissue	c.121G > T	–	12.2% (506/3,739)
P34	TMU	60	F	L	Extraconal	13	FFPE tissue	c.121G > T	–	9.3% (366/3,555)
P35	TMU	51	M	L	Extraconal	14	FFPE tissue	c.121G > T	–	15.3% (1,174/7,449)
P36	TMU	55	F	L	Intraconal	10	FFPE tissue	c.121G > T	–	16.4% (307/1,806)
P37	TMU	63	F	R	Extraconal	10	FFPE tissue	c.121G > T	–	7.7% (578/6,226)
P38	TMU	44	F	R	Intraconal	16	FFPE tissue	c.121G > T	–	14.3% (1,672/9,121)
P39	TMU	57	M	R	Extraconal	34	Frozen tissue	c.121G > T	–	7.5% (559/6,328)
P40	TMU	18	M	L	Extraconal	13	Frozen tissue	Negative	–	0% (0/5,122)
P41	TMU	47	F	L	Extraconal	22	Frozen tissue	c.121G > T	–	10.0% (541/4,786)
P42	TMU	36	M	R	Extraconal	20	Frozen tissue	c.121G > T	–	11.8% (713/5,370)
P43	TMU	48	F	R	Extraconal	20	Frozen tissue	c.121G > T	–	9.8% (681/5,925)
							CD31-positive cells		–	34.2% (810/2,285)
							CD31-negative cells		–	2.0% (188/5,733)
							Blood	Negative	–	0% (0/9,200)
P44	TMU	51	F	R	Intraconal	13	Frozen tissue	c.121G > T	–	15.4% (1,605/8,226)
							CD31-positive cells		–	38.3% (567/1,470)
							CD31-negative cells		–	2.2% (18/792)
							Blood	Negative	–	0% (0/6,269)

Targeted deep sequencing columns indicate read depth, and ddPCR columns indicate number of droplets. Calculated MAF (mutation allele frequency) is also depicted in these columns. MAFs in Targeted deep sequencing columns were calculated from the number of mutation reads/total reads at that locus. MAFs in ddPCR columns were calculated from the number by counting droplets that contain mutation, mutation + wild-type, and wild-type amplimers. For simplicity, the ratio of mutation amplimer containing droplets/all amplimer containing droplets is shown

*ddPCR* droplet digital polymerase chain reaction, *UT* The University of Tokyo, *TMU* Tokyo medical university, *F* female, *M* male, *L* left, *R* right, *FFPE* formalin-fixed paraffin-embedded, *NA* not available

*GenBank: NM_002060.3

Dash (–) indicates that the assay was not performed

GJA4, which is encoded by *GJA4*, is an isoform of connexin, which is a member of a family of transmembrane proteins. Connexins oligomerize to form channels called hemichannels, which connect the cytoplasm with the extracellular space, and gap junctions, which connects the cytoplasm of adjacent cells [26]. The connexin family has 21 members in humans; GJA4 is expressed in the endothelium and smooth muscle of blood vessels [11]. Connexins contain a pore-forming domain, which includes four transmembrane domains, and a carboxy-terminal domain, the cytoplasmic tail of which is frequently phosphorylated [27]. *GJA4* p.Gly41Cys is located at the extracellular end of transmembrane domain 1 (Fig. 1c). *GJA4 p.Gly41Cys* occurred at a position highly conserved among species and moderately conserved among connexin isoforms in humans (Fig. 1d and Supplemental Fig. S3).

### Detection of *GJA4* c.121G > T (p.Gly41Cys) in lesional endothelial cells

**Fig. 2:**
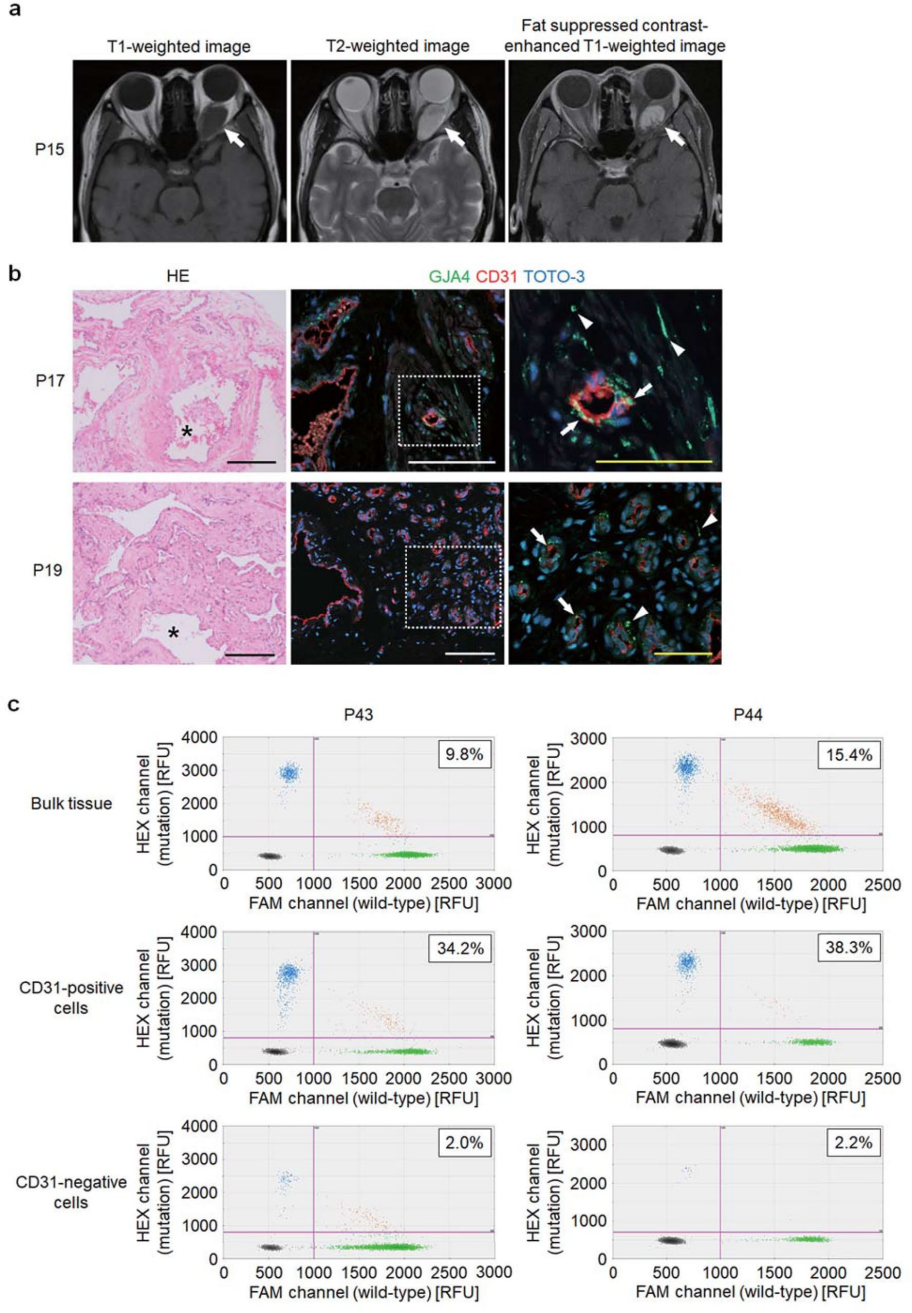
GJA4 expression and *GJA4* c.121G > T (p.Gly41Cys) mutation allele frequency in OCVM endothelial cells. **a** Representative MRI images of OCVM lesions (images of P15). **b** Hematoxylin and eosin (HE) staining (left) and immunostaining images for GJA4, endothelial cell marker CD31, and nuclear marker TOTO®-3 (middle and right) of sections of OCVM tissue from P17 and P19. Asterisks indicate vascular channels. White dotted lines indicate the positions of magnified panels (right). Arrows indicate GJA4 expression in endothelial cells, and arrowheads indicate GJA4 expression in non-endothelial cells. Black, white, and yellow scale bars indicate 200, 100, and 50 μm respectively. **c ** Results of the quantification of mutation allele abundance using ddPCR in bulk tissue, CD31-positive and negative cells, from prospectively collected samples from 2 OCVMs (P43 and P44). Each dot represents a droplet, with blue being mutation positive, green being wild-type positive, and orange being positive for both. The boxed number is the fractional abundance of mutation allele in each sample

OCVM appears as a well-defined homogeneous mass isointense to muscle in T1-weighted images and hyperintense to fat and brain in T2-weighted images on magnetic resonance imaging (MRI) (Fig. 2a). The lesions contain varying degrees of contrast enhancement from patchy and heterogeneous to uniform, depending on the phase of imaging, suggesting contrast filling over time [2]. Histologically, OCVM is characterized by multiple dilated vascular channels covered by endothelial cells surrounded by abundant fibrous stroma (Fig. 2b). The stroma contains sparse smooth muscle bundles. Ki-67, a marker of cellular proliferation is negative (Supplemental Fig. S4). Immunohistochemistry with an antibody specific for GJA4 N-terminal, which does not include the position of the mutation, showed scattered expression of GJA4 in endothelial cells and the stroma.

Somatic mutations identified in other types of vascular malformations were highly enriched in endothelial cells within the lesions [19, 22, 28, 29]. To determine whether *GJA4* c.121G > T (p.Gly41Cys) is also enriched in endothelial cells in OCVM, we prospectively collected samples from 3 freshly resected vascular anomalies (2 OCVMs and one conjunctival capillary hemangioma), and obtained CD31-positive and negative cells (endothelial and non-endothelial cells, respectively), in addition to bulk tissue samples using MACS, and performed ddPCR. First, we performed ddPCR using bulk tissue samples. *GJA4* c.121G > T (p.Gly41Cys) was detected in the 2 OCVM samples (MAF = 9.8% and 15.4%, respectively); the conjunctival capillary hemangioma sample was negative for the mutation (Table S2 in the Data Supplement). Then, we performed ddPCR using genomic DNA extracted from CD31-positive and negative cells from the 2 OCVM samples. Results revealed that the MAFs of *GJA4* c.121G > T (p.Gly41Cys) were higher in CD31-positive cells than bulk tissue in both 2 OCVM samples (MAFs for CD31-positive cells in the 2 samples were 34.2% and 38.2%, respectively) (Fig. 2c and Supplemental Table S3). MAFs for CD31-negative cells were very low in the 2 samples (2.0% and 2.2%, respectively). These findings suggest that endothelial cells predominantly contain *GJA4* c.121G > T (p.Gly41Cys) in OCVM tissue samples.

### *GJA4* c.121G > T (p.Gly41Cys) leads to the formation of hyperactive hemichannels

Various mutations have been identified in several connexin isoforms [30]. These mutations are involved in disease pathogenesis by affecting the properties of connexin channels in both a loss- and gain-of-function manner [31]. We hypothesized that *GJA4* c.121G > T (p.Gly41Cys) contributes to GJA4 channel abnormality and performed whole-cell voltage clamp analysis, a standard electrophysiological method to evaluate channel conductance [32], in *Xenopus* oocytes to measure hemichannel and transjunctional currents.

**Fig. 3:**
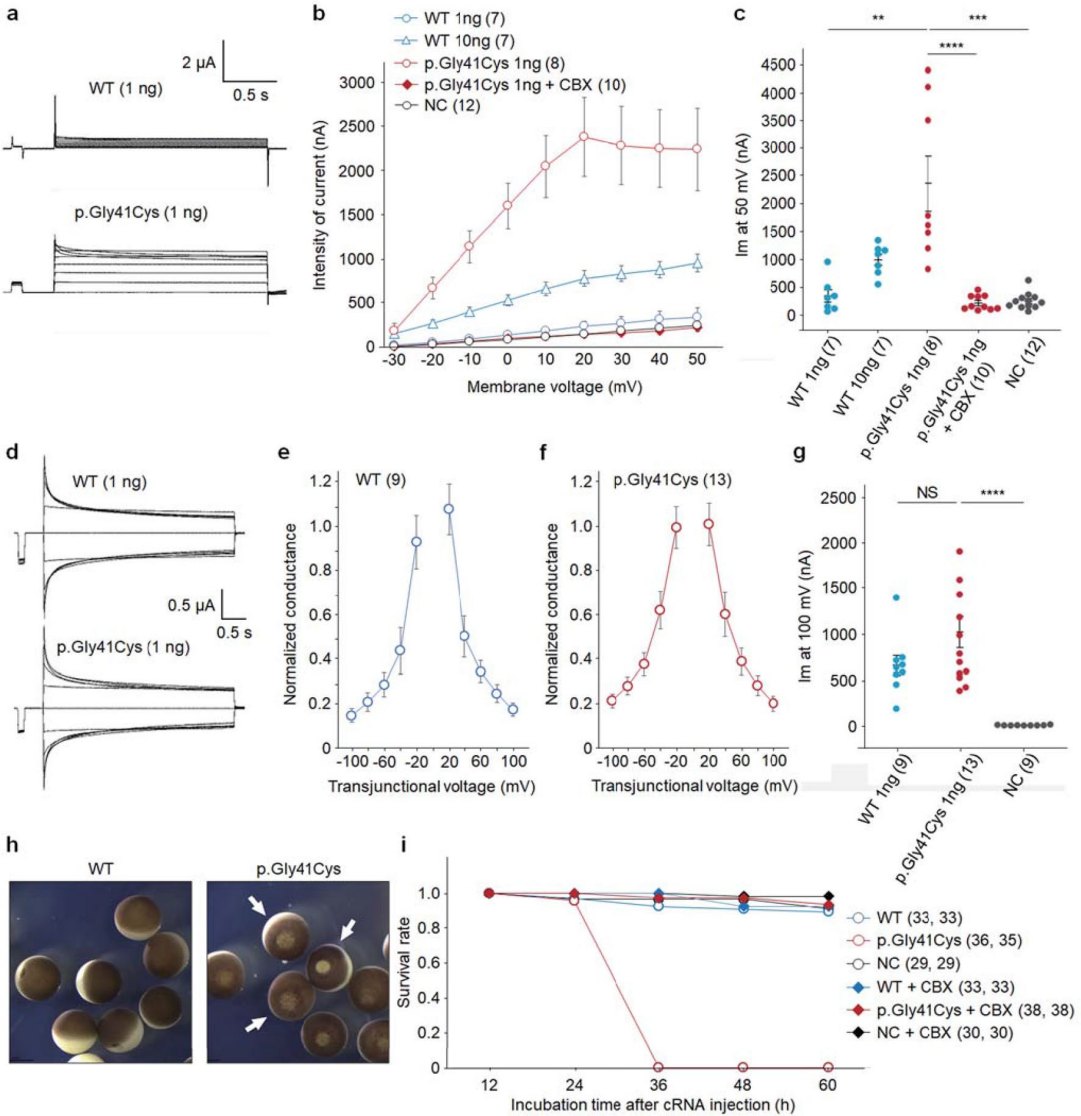
GJA4 p.Gly41Cys increases GJA4 hemichannel activity. **a** Representative whole-cell voltage clamp hemichannel-recording of *Xenopus* oocytes injected with 1 ng of *GJA4* WT or *GJA4* p.Gly41Cys cRNA. **b** Intensity of hemichannel currents at the steady state plotted as a function of membrane voltage. The number of oocytes analyzed is given in parentheses. Data are shown as means ± SEM. **c** Comparison of hemichannel currents at a steady state of 50 mV membrane voltage. Data are shown as means ± SEM. **d** Representative whole-cell voltage clamp gap junction-recording of *Xenopus* oocytes injected with 1 ng *GJA4* WT or *GJA4* p.Gly41Cys cRNA. **e** A plot of normalized steady state junctional conductance versus transjunctional voltage (Gj–Vj plot) for GJA4 WT. The number of oocytes analyzed is given in parentheses. Data are shown as means ± SEM. **f** Plots of normalized steady state junctional conductance versus transjunctional voltage (Gj–Vj plot) for GJA4 p.Gly41Cys. The number of oocytes analyzed is given in parentheses. Data are shown as means ± SEM. **g** Comparison of transjunctional current at a steady state of 100 mV membrane voltage. Data are shown as means ± SEM. *P* value was calculated using a two-tailed *t* test. **h** Images showing the deleterious effect of GJA4 on oocytes (arrows) injected with *GJA4* c.121G > T (p.Gly41Cys) compared with GJA4 WT 36 h after cRNA injections. **i** Plots of survival rates of oocytes injected with *GJA4* WT and c.121G > T (p.Gly41Cys). The number of oocytes analyzed is given in parentheses. The averages of two replicated experiments were plotted. *P* values were calculated using a two-tailed *t* test. NS not significant; *p < 0.05; **p < 0.01; ***p < 0.001; ****p < 0.0001

First, we measured hemichannel currents in single oocytes injected with cRNA from *GJA4* WT and *GJA4* c.121G > T (p.Gly41Cys). Membrane currents were detected in both GJA4 WT and GJA4 p.Gly41Cys oocytes (Fig. 3a). In GJA4 WT oocytes, current intensity increased depending on the amount of *GJA4* WT cRNA, and in both GJA4 WT and GJA4 p.Gly41Cys oocytes, current intensity increased upon increasing membrane voltage (Fig. 3b). Compared with current values of + 50 mV in the control, membrane current value in GJA4 p.Gly41Cys was significantly higher than that in GJA4 WT (Fig. 3c). Moreover, the hemichannel activity in GJA4 p.Gly41Cys oocytes was inhibited by 100 μM CBX, which is a non-specific inhibitor of hemichannels and gap junctions [33], indicating an increase in current derived from hemichannels formed of GJA4 p.Gly41Cys. These findings suggest that GJA4 p.Gly41Cys forms hemichannels on the oocyte plasma membrane and increases its activity.

Second, we measured transjunctional current in paired oocytes. Transjunctional currents were induced in GJA4 WT and GJA4 p.Gly41Cys oocytes (Fig. 3d), which displayed quick time-dependent inactivation in response. The Gj–Vj plot of GJA4 WT displayed sensitivity to transjunctional voltage; gap junction channels closed in response to the voltage imposed across the junctional membrane (Fig. 3e). The Gj–Vj plot of GJA4 p.Gly41Cys also showed sensitivity to transjunctional voltage, which was similar to that observed in the GJA4 WT gap junction channel (Fig. 3f). These findings indicate that the gap junction channel formed by GJA4 p.Gly41Cys has similar gating properties to those induced by GJA4 WT. Compared with the + 60 mV current at the steady state, the difference in transjunctional current values between GJA4 WT and GJA4 p.Gly41Cys was not significant, although currents induced by both GJA4 WT and GJA4 p.Gly41Cys were significantly higher than that in normal control (NC) (Fig. 3g). These data revealed that GJA4 p.Gly41Cys has only a minor impact on the formation and function of the gap junction channel. Thus, *GJA4* c.121G > T (p.Gly41Cys) is a gain-of-function mutation, which leads to the formation of hyperactive hemichannels.

To further investigate the effect of GJA4 p.Gly41Cys hemichannels on oocytes, we evaluated oocytes injected with *GJA4* WT and *GJA4* c.121G > T (p.Gly41Cys) cRNAs after ≤ 60 h. Oocytes injected with *GJA4* p.Gly41Cys cRNA began dying within 36 h after the injection (Fig. 3h). CBX rescued this phenotype, with GJA4 p.Gly41Cys expressing oocytes surviving until 60 h after the cRNA injection, indicating that GJA4 p.Gly41Cys deleteriously affects oocytes by forming hyperactive hemichannels (Fig. 3i).

### GJA4 p.Gly41Cys dysregulates endothelial cell-function in vitro

**Fig. 4:**
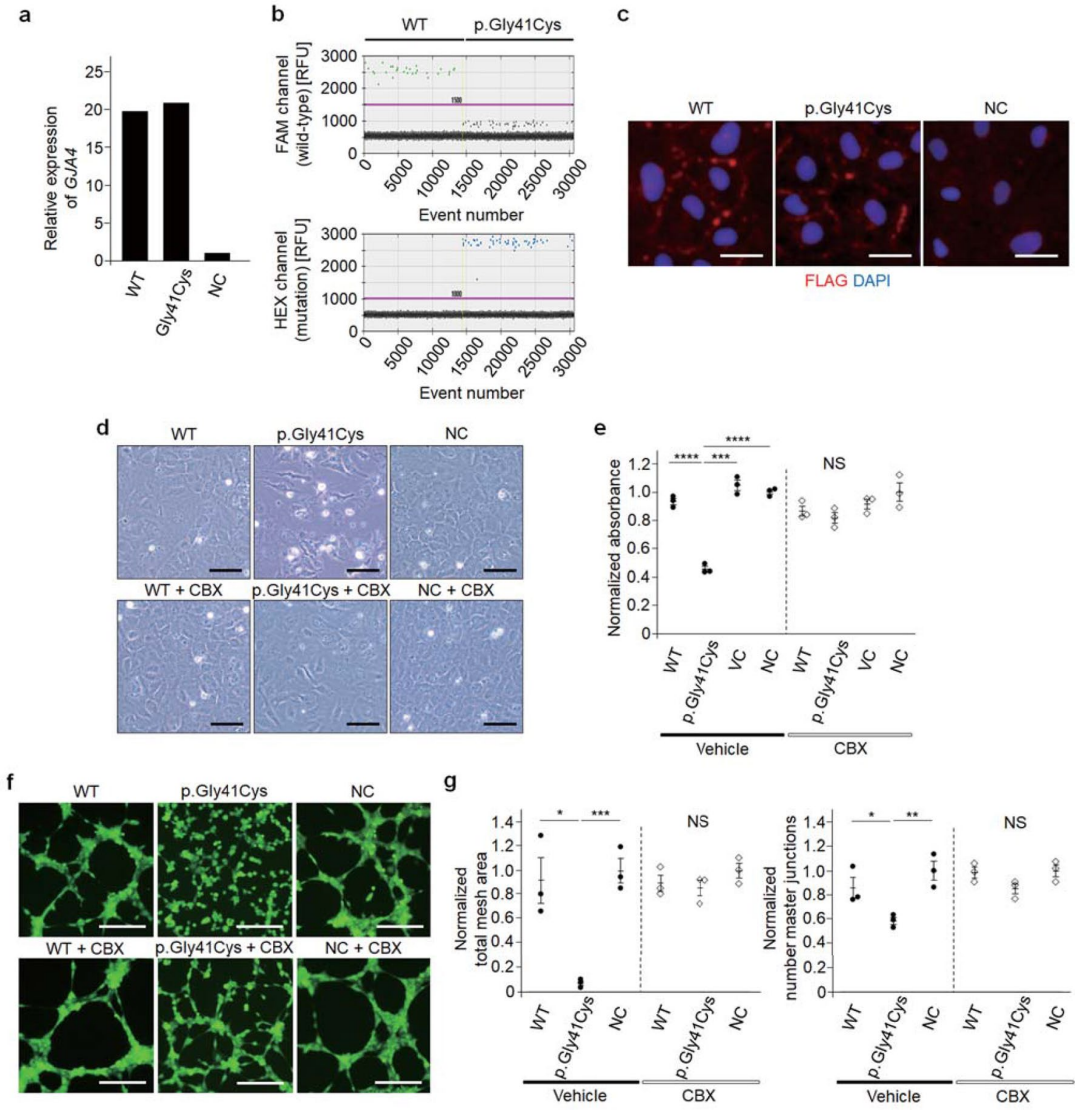
GJA4 p.Gly41Cys dysregulates endothelial cell-function, which is rescued by a connexin inhibitor. **a** qPCR analyses of *GJA4* mRNA in HUVECs transfected with *GJA4* WT or *GJA4* c.121G > T (p.Gly41Cys). **b** ddPCR analysis of *GJA4* cDNA in HUVECs transfected with *GJA4* WT or *GJA4* c.121G > T (p.Gly41Cys). Each dot represents a droplet, with blue being mutation positive, and green being wild-type positive. **c** Immunostaining for FLAG and nuclear marker DAPI of HUVECs transfected with *GJA4* WT-FLAG, *GJA4* c.121G > T (p.Gly41Cys)-FLAG, or normal control. Scale bars, 15 μm. **d** Representative image of HUVECs transfected with *GJA4* WT, *GJA4* c.121G > T (p.Gly41Cys), or normal control, and efficacy of CBX. Scale bars, 50 μm. **e** Quantification of cell viability of HUVECs transfected with *GJA4* WT, *GJA4* c.121G > T (p.Gly41Cys), vector control, and normal control using the MTT assay. **f** Representative images of tube formation assay of HUVECs transfected with *GJA4* WT, *GJA4* c.121G > T (p.Gly41Cys), or normal control, and the efficacy of CBX. Scale bars, 200 μm. **g** Quantification of tube formation in HUVECs transfected with *GJA4* WT, *GJA4* c.121G > T (p.Gly41Cys), and normal control by total mesh area (left) and number master junctions (right). *P* values were calculated using a two-tailed *t* test. NS, not significant; *p < 0.05; **p < 0.01; ***p < 0.001; ****p < 0.0001

Endothelial cell-predominant mutation distribution in OVCM tissues and physiological influences confirmed in *Xenopus* oocytes suggest a role for the mutation and the resulting hemichannel activation in OVCM. To evaluate the impact of GJA4 p.Gly41Cys and functional rescue of hyperactive hemichannels with CBX on human vascular endothelial cells, we comparatively analyzed cell morphology, viability, and tube formation capacity using HUVECs overexpressing GJA4 WT and GJA4 p.Gly41Cys, prepared through retroviral gene transduction. Retroviral gene transduction was validated with qPCR and ddPCR (Fig. 4a, b). Immunofluorescence analyses of FLAG-tagged GJA4 WT and GJA4 p.Gly41Cys transduced cells revealed cell-junction-specific localization (Fig. 4c). GJA4 p.Gly41Cys overexpression disrupted endothelial cell-specific morphology compared with GJA4 WT overexpression. This deformation was partially rescued by CBX (20 μM) (Fig. 4d). Cell viability, assessed using the MTT assay, significantly decreased only in GJA4 p.Gly41Cys overexpressing HUVECs and was rescued by CBX (Fig. 4e). Results from the in vitro tube formation assay, in which spontaneous vascular tube formation on a basement membrane matrix is quantified as total mesh area and number of master junctions [34], revealed that tube formation is disrupted in HUVECs overexpressing GJA4 p.Gly41Cys; this phenotype was rescued with CBX (Fig. 4f, g). Thus, *GJA4* c.121G > T (p.Gly41Cys) affects the characteristics of endothelial cells by increasing hemichannel activity; inhibition of hyperactive hemichannels can be used for treating the abnormal phenotypes of endothelial cells induced by *GJA4* c.121G > T (p.Gly41Cys).

## Discussion

In this study, we identified a somatic *GJA4* mutation (c.121G > T [p.Gly41Cys]) in most tissue samples of OCVM, suggesting *GJA4* as a driver of OCVM. The MAF of *GJA4* c.121G > T (p.Gly41Cys) was higher in CD31-positive cells and GJA4 expression was detected in OCVM tissues, including endothelial cells. Results from electrophysiological studies revealed that GJA4 p.Gly41Cys forms a hyperactive hemichannel, hence classified as a gain-of-function mutation which adversely affects the viability and function of HUVECs. These phenotypes were rescued by CBX, a non-specific inhibitor of hemichannels and gap junctions. Thus, *GJA4* c.121G > T (p.Gly41Cys) dysregulates the biological activities of endothelial cells and may be involved in the pathogenesis of OCVM.

The MAFs of *GJA4* c.121G > T (p.Gly41Cys) were relatively low (4.6–16.4%), possibly due to the cellular heterogeneity of vascular malformations; here, we identified a higher MAF in the endothelial cell-enriched fraction using MACS. This finding suggests that *GJA4* c.121G > T (p.Gly41Cys) occurs and resides within endothelial cells in OCVM lesions. Studies on other vascular malformations, such as brain arteriovenous malformation (MIM: 108010), extracranial arteriovenous malformation, cerebral cavernous malformation, and capillary malformation-arteriovenous malformation syndrome (MIM: 608354), also demonstrated an enrichment of respective gene mutations in lesional endothelial cells [19, 22, 28, 29]. Considering these findings, we speculate that the occurrence of gene mutations and the subsequent dysfunction within endothelial cells would be common primary events in the pathogenesis of vascular malformations.

GJA4 is a connexin isoform expressed in the vascular system. Electrical and molecular communication across hemichannels and gap junctions are essential for coordinating vascular behavior [11]. Studies have reported essential roles, such as angiogenesis, vascular remodeling, and vascular permeability, for GJA4 in the vascular system [35–37]. Within the vascular wall, GJA4 is expressed preferentially in endothelial cells [38, 39]. GJA4 is also involved in the regulation of endothelial cell cycle to promote arterial gene expression [40]. GJA4- and GJA5- (another connexin expressed in the vascular system) deficient mice exhibited abnormal vascular channels, with these channels coalescing into a cavernous, endothelium-lined blood pool [41]. These studies strongly suggest a critical role for GJA4 in vascular development. In OCVM, *GJA4* c.121G > T (p.Gly41Cys) led to endothelial cell dysfunction, such as abnormal cell morphology, lower cellular viability, and decreased tube formation, which indicates that *GJA4* c.121G > T (p.Gly41Cys) plays a role in the vascular phenotype of OCVM. An identical mutation, c.121G > T (p.Gly41Cys) in *GJA4* was reported in hepatic hemangiomas and cutaneous venous malformations [42]. Together with these findings, our data underline the significance of this mutation as a potential driver gene mutation for a broad range of vascular malformations.

Studies have identified numerous mutations in connexin genes in various human diseases affecting disease pathogenesis in both a gain- and loss-of-function manner [30]. Here we demonstrated that GJA4 p.Gly41Cys leads to the formation of a hyperactive hemichannel. The location of the mutation at the extracellular end of transmembrane domain 1 is consistent with findings that most mutations leading to the formation of hyperactive hemichannels are present in the N-terminus, extracellular loop 1, and transmembrane domains 1 and 2 [43]. Hyperactive hemichannels are associated with connexins and diseases, including keratitis-ichthyosis-deafness syndrome (MIM: 148210) (GJB2 [Cx26] [MIM: 121011]), Charcot–Marie–Tooth disease (MIM: 302800) (GJB1 [Cx32] [MIM: 304040]), oculodentodigital dysplasia (MIM: 164200) (GJA1 [Cx43] [MIM: 121014]), and cataract (MIM: 116200) (GJA8 [Cx50] [MIM: 600897]), although their etiologies are unknown [44–47]. Our findings reveal that hyperactive hemichannels adversely impact endothelial cell biology, which may contribute to the pathophysiology of OCVM.

CBX rescuing the endothelial cell dysfunction induced by GJA4 p.Gly41Cys confirm that the dysfunction was caused by a hyperactive hemichannel originating due to GJA4 p.Gly41Cys. Thus, we propose CBX as a therapeutic agent in OCVM, which is a non-specific, broadly-acting connexin inhibitor, extensively used to study connexin function in vitro and in vivo.[48, 49] CBX has been clinically approved and widely used for treating gastropeptic, esophageal, and oral ulcers for more than a decade [50]. As known candidates for targeted treatment, molecular inhibitors such as MEK inhibitor and mTOR inhibitor have been used for some types of vascular malformations, because most vascular malformations are caused by genetic mutations that lead to the hyperactivity of RAS/MAPK/ERK or PI3K/AKT/mTOR pathways [9]. Therefore, although clarifying the association between *GJA4* c.121G > T (p.Gly41Cys) and established signaling pathways is warranted, the hyperactive hemichannel should be considered as an additional therapeutic target for vascular malformations.

## Supplementary Information

Below is the link to the electronic supplementary material.

Supplementary file1 (DOCX 4119 kb)

## Data Availability

The targeted deep sequencing datasets are available in JGA under accession number JGAS000325. Additional data that support the findings of this study are available from the corresponding author on reasonable request.

## Abbreviations

AKT: Protein kinase B
BWA: Burrows–wheeler aligner
CADD: Combined annotation dependent depletion
CBX: Carbenoxolone
CCM: Cerebral cavernous malformation
cDNA: Complementary DNA
cRNA: Complementary RNA
ddPCR: Droplet digital PCR
ERK: Extracellular signal-regulated kinase
FFPE: Formalin-fixed paraffin-embedded
HUVEC: Human umbilical vein endothelial cell
ISSVA: International society for the study of vascular anomalies
JGA: Japanese genotype–phenotype archive
MACS: Magnetic-activated cell sorting
MAF: Mutation allele frequency
MAPK: Mitogen-activated protein kinase
MRI: Magnetic resonance imaging
mTOR: Mammalian target of rapamycin
MTT: 3-[4,5-Dimethylthiazol-2-yl]-2,5 diphenyl tetrazolium bromide
NC: Normal control
OCVM: Orbital cavernous venous malformation
PBS: Phosphate-buffered saline
PI3K: Phosphatidylinositol 3-kinase
qPCR: Quantitative PCR
TBS-T: Tris-buffered saline with Tween 20
TMU: Tokyo medical university
UT: The University of Tokyo
VH: Vertebral hemangioma
WT: Wild-type
