# Supplementary material for: Somatic GJA4 gain-of-function mutation in orbital cavernous venous malformations
Source: Angiogenesis. 2022 Jul 29;26(1):37–52. doi: 10.1007/s10456-022-09846-5 (PMC9908695; doi:10.1007/s10456-022-09846-5)
Supplement: Supplementary file 1 — Supplementary file1 (DOCX 4119 kb) [file 10456_2022_9846_MOESM1_ESM.docx]

**Supplementary Material for:**

**Somatic *GJA*4 gain-of-function mutation in orbital cavernous venous malformations**

Hiroki Hongo^1^, Satoru Miyawaki^1,*^ , Yu Teranishi^1^, Jun Mitsui^2^, Hiroto Katoh^3^, Daisuke Komura^3^, Kinya Tsubota^4^, Takashi Matsukawa^2^, Masakatsu Watanabe^5^, Masakazu Kurita^6^, Jun Yoshimura^7^, Shogo Dofuku^1^, Kenta Ohara^1^, Daiichiro Ishigami^1^, Atsushi Okano^1^, Motoi Kato^6^, Fumihiko Hakuno^8^, Ayaka Takahashi^8^, Akiko Kunita^9^, Hiroyuki Ishiura^10^, Masahiro Shin^1^, Hirofumi Nakatomi^1^, Toshitaka Nagao^11^, Hiroshi Goto^4^, Shin-Ichiro Takahashi^8^, Tetsuo Ushiku^9^, Shumpei Ishikawa^3^, Mutsumi Okazaki^6^, Shinichi Morishita^7^, Shoji Tsuji^2,12^, Nobuhito Saito^1^

^1^Department of Neurosurgery, Faculty of Medicine, The University of Tokyo, Tokyo, Japan

^2^Department of Molecular Neurology, Graduate School of Medicine, The University of Tokyo, Tokyo, Japan

^3^Department of Preventive Medicine, Graduate School of Medicine, The University of Tokyo, Tokyo, Japan

^4^Department of Ophthalmology, Tokyo Medical University, Tokyo, Japan

^5^Laboratory of Pattern Formation, Graduate School of Frontier Biosciences, Osaka University, Suita, Osaka, Japan

^6^Department of Plastic and Reconstructive Surgery, The University of Tokyo, Tokyo, Japan

^7^Department of Computational Biology and Medical Sciences, Graduate School of Frontier Sciences, The University of Tokyo, Kashiwa, Chiba, Japan

^8^Department of Animal Resource Sciences, Graduate School of Agriculture and Life Sciences, The University of Tokyo, Tokyo, Japan

^9^Department of Pathology, Graduate School of Medicine, The University of Tokyo, Tokyo, Japan

^10^Department of Neurology, Faculty of Medicine, The University of Tokyo, Tokyo, Japan

^11^Department of Anatomic Pathology, Tokyo Medical University, Tokyo, Japan

^12^Institute of Medical Genomics, International University of Health and Welfare, Narita, Chiba, Japan

***Corresponding author:**

Satoru Miyawaki, MD, PhD, Department of Neurosurgery, Faculty of Medicine, The University of Tokyo, 7-3-1 Hongo, Bunkyo-ku, Tokyo 113-8655, Japan

Tel.: +81-3-5800-8853; Fax: +81-3-5800-8655; E-mail: [smiya-nsu@m.u-tokyo.ac.jp](mailto:smiya-nsu@m.u-tokyo.ac.jp)

**Journal: Angiogenesis**

This document contains the following material:

Supplemental Figures S1-4.

Supplemental Tables S1-3.

**Supplemental Figures**


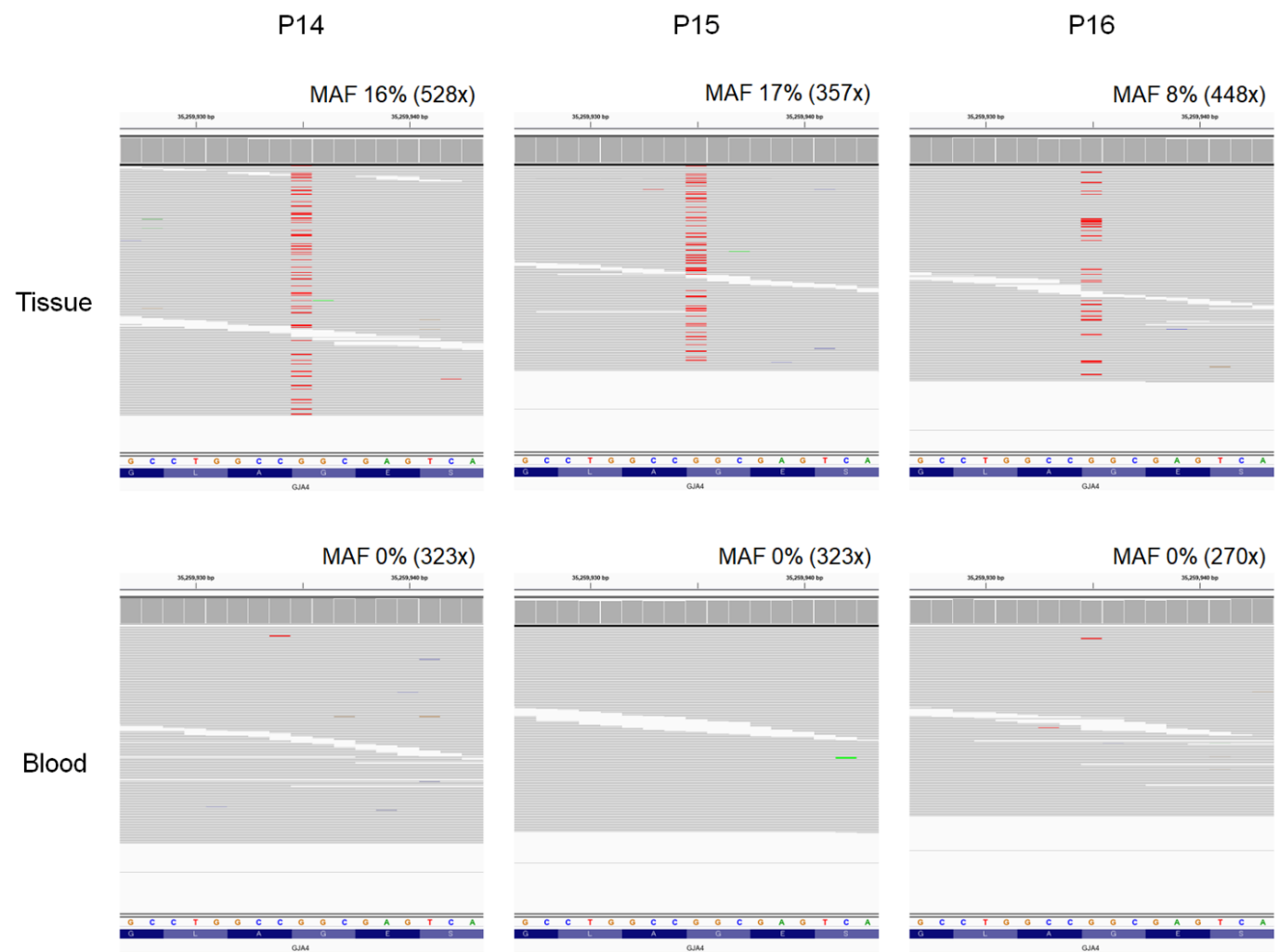
 **Fig. S1 Integrative Genomic Viewer screenshots showing targeted deep sequencing reads of 3 orbital cavernous venous malformation (OCVM) participants across the area of *GJA4* c.121G>T (p.Gly41Cys).** Upper and lower columns show images of tissue samples and blood samples, respectively. Variant nucleotides (T) are shown in red. The reference nucleotide and amino acid sequences are at the bottom. Participant IDs are shown above. The mutation allele frequencies (MAFs) and sequencing coverages at the position are shown above each image


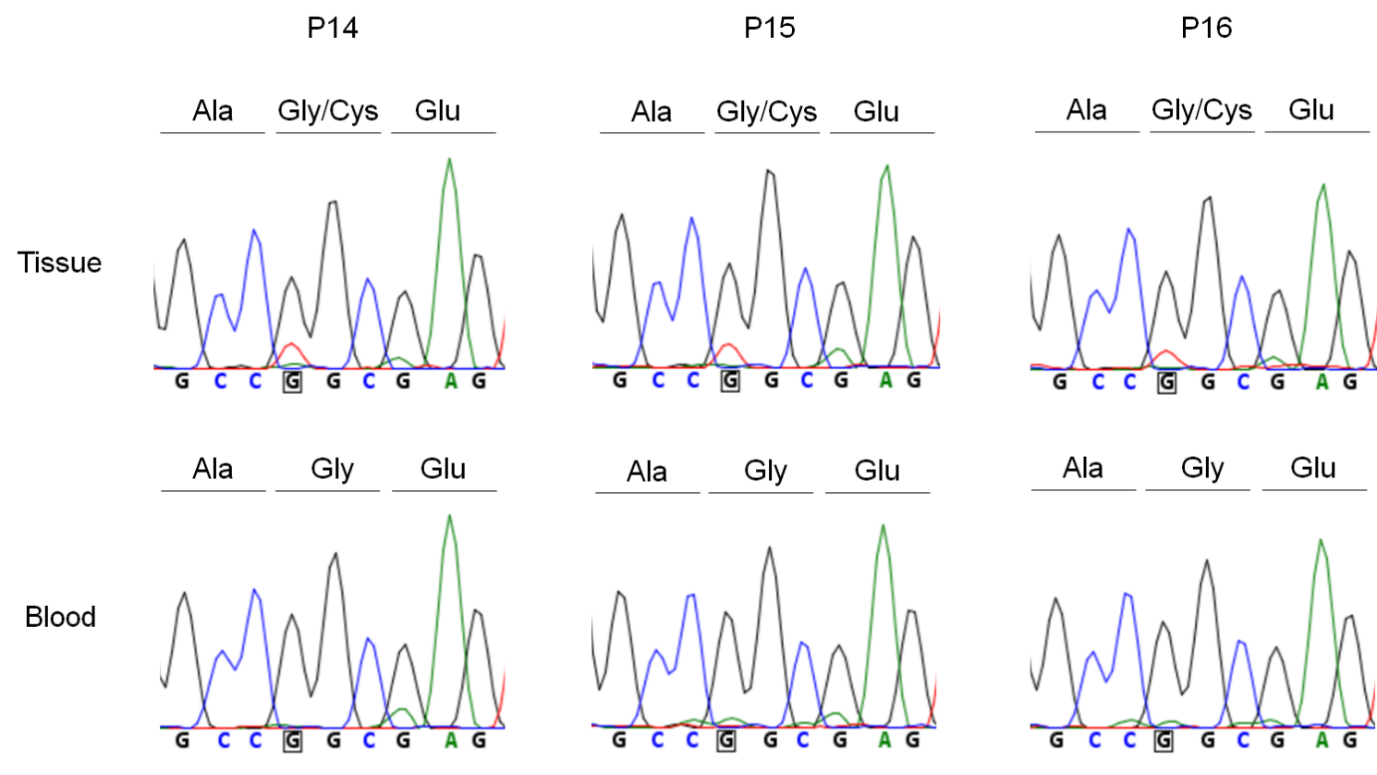


**Fig. S2 Sanger sequencing chromatograms showing *GJA4* sequences of 3 OCVM participants in the discovery cohort.** Each curve represents a trace of a signal intensity; black, blue, green, and red represent G (guanine), C (cytosine), A (adenine), and T (thymine), respectively. Boxed characters represent nucleotides at the position of the *GJA4* mutation (c.121G>T). Upper and lower columns show chromatograms of tissue samples and blood samples, respectively. Participant IDs are shown above. Amino acid sequences are shown above each chromatogram


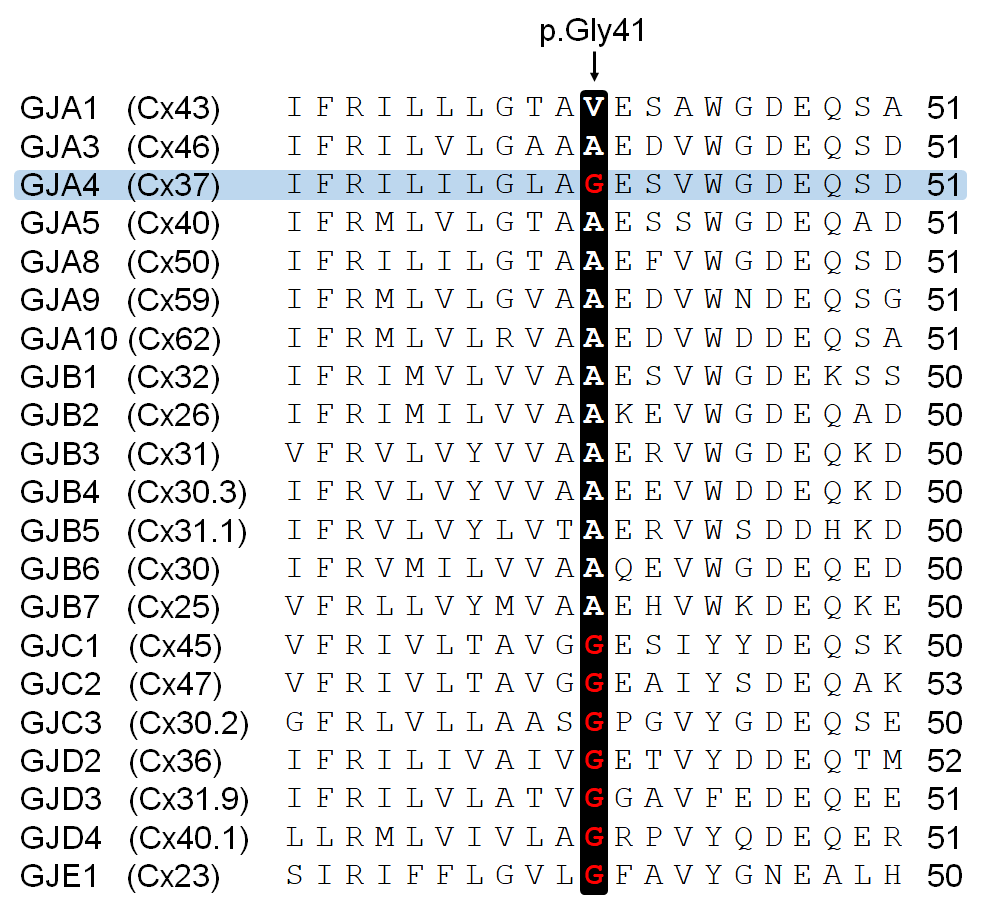


**Fig. S3 Conservation of amino acid position among connexin families in humans.** Sequence alignments, CLUSTALW. Genbank numbers are as follows: GJA1, NP_000156.1; GJA3, NP_068773.2; GJA4, NP_002051.2; GJA5, NP_005257.2; GJA8, NP_005258.2; GJA9, NP_110399.2; GJA10, NP_115991.1; GJB1, NP_000157.1; GJB2, NP_003995.2; GJB3, NP_001005752.1; GJB4, NP_694944.1; GJB5, NP_005259.1; GJB6, NP_001103691.1; GJB7, NP_940970.1; GJC1, NP_001073852.1; GJC2, NP_065168.2; GJC3, NP_853516.1; GJD2, NP_065711.1; GJD3, NP_689343.3; GJD4, NP_699199.2; GJE1, NP_001345339.1


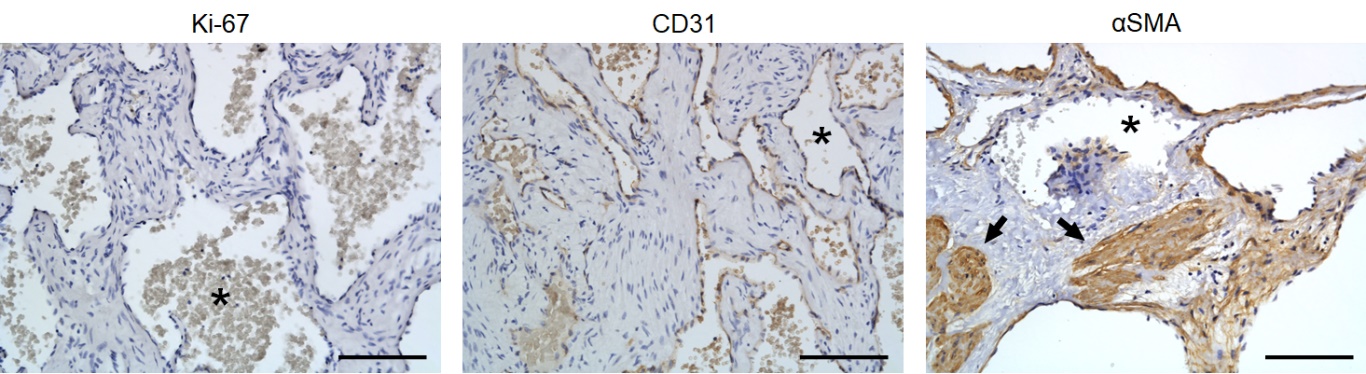


**Fig. S4 Immunostaining of a section of OCVM for Ki-67, endothelial cell marker CD31, and smooth muscle cell marker αSMA.** Tissue is from Participant 15. Asterisks indicate vascular channels. Arrows indicate smooth muscle bundles. Scale bars, 100 μm

**Supplemental Tables**

**Table S1 Detection of *GJA4* c.121G>T (p.Gly41Cys) in the discovery cohort**

| Participant ID | diagnosis | targeted sequencing (tissue) | | | targeted sequencing (blood) | | | ddPCR (tissue) | | | ddPCR (blood) | | |
| --- | --- | --- | --- | --- | --- | --- | --- | --- | --- | --- | --- | --- | --- |
|  |  | MUT reads | WT reads | tissue MAF | MUT reads | WT reads | blood MAF | MUT droplets | WT droplets | tissue MAF | MUT droplets | WT droplets | blood MAF |
| P1 | CCM | NA | NA | 0 | NA | NA | 0 | 0 | 6577 | 0 | 0 | 4903 | 0 |
| P2 | CCM | NA | NA | 0 | NA | NA | 0 | 0 | 5154 | 0 | 0 | 6520 | 0 |
| P3 | CCM | NA | NA | 0 | NA | NA | 0 | 0 | 5190 | 0 | 0 | 5615 | 0 |
| P4 | CCM | NA | NA | 0 | NA | NA | 0 | 0 | 3594 | 0 | 0 | 5486 | 0 |
| P5 | CCM | NA | NA | 0 | NA | NA | 0 | 0 | 5251 | 0 | 0 | 4720 | 0 |
| P6 | CCM | NA | NA | 0 | NA | NA | 0 | 0 | 3417 | 0 | 0 | 5806 | 0 |
| P7 | CCM | NA | NA | 0 | NA | NA | 0 | 0 | 5382 | 0 | 0 | 3994 | 0 |
| P8 | CCM | NA | NA | 0 | NA | NA | 0 | 0 | 3630 | 0 | 0 | 5692 | 0 |
| P9 | CCM | NA | NA | 0 | NA | NA | 0 | 0 | 2948 | 0 | 0 | 6337 | 0 |
| P10 | CCM | NA | NA | 0 | NA | NA | 0 | 0 | 4011 | 0 | 0 | 5373 | 0 |
| P11 | CCM | NA | NA | 0 | NA | NA | 0 | 0 | 4373 | 0 | 0 | 5959 | 0 |
| P12 | CCM | NA | NA | 0 | NA | NA | 0 | 0 | 3593 | 0 | 0 | 5303 | 0 |
| P13 | VH | NA | NA | 0 | NA | NA | 0 | 0 | 5594 | 0 | 0 | 4966 | 0 |
| P14 | OCVM | 75 | 447 | 0.168 | 0 | 282 | 0 | 591 | 2880 | 0.157 | 0 | 4260 | 0 |
| P15 | OCVM | 54 | 322 | 0.168 | 0 | 279 | 0 | 975 | 4568 | 0.153 | 0 | 3832 | 0 |
| P16 | OCVM | 35 | 403 | 0.087 | 1 | 228 | 0.004 | 1152 | 7496 | 0.094 | 0 | 5426 | 0 |

CCM, cerebral cavernous malformation; VH, vertebral hemangioma; OCVM, orbital cavernous venous malformation

ddPCR, droplet digital polymerase chain reaction

MUT, mutation; WT, wild-type, MAF, mutation allele frequency

NA, not available

**Table S2 ddPCR results for *GJA4* c.121G>T (p.Gly41Cys) of samples in discovery and validation cohorts**

| Participant ID | diagnosis | cohort | frozen tissue DNA | | | FFPE tissue DNA | | | FFPE tissue DNA | | | FFPE tissue DNA | | | definitive MAF | mutation status | blood DNA | | | mutation status |
| --- | --- | --- | --- | --- | --- | --- | --- | --- | --- | --- | --- | --- | --- | --- | --- | --- | --- | --- | --- | --- |
|  |  |  |  |  |  | (isolation 1) | | | (isolation 2) | | | (isolation 3) | | |  |  |  |  |  |  |
|  |  |  | MUT droplets | WT droplets | MAF | MUT droplets | WT droplets | MAF | MUT droplets | WT droplets | MAF | MUT droplets | WT droplets | MAF |  |  | MUT droplets | WT droplets | MAF |  |
| P14 | OCVM | UT | 591 | 2880 | **0.157** | 1960 | 9696 | 0.113 |  |  |  |  |  |  | 0.157 | Positive | 0 | 4260 | 0 | Negative |
| P15 | OCVM | UT | 975 | 4568 | **0.153** | NA | | |  |  |  |  |  |  | 0.153 | Positive | 0 | 3832 | 0 | Negative |
| P16 | OCVM | UT | 1152 | 7496 | **0.094** | NA | | |  |  |  |  |  |  | 0.094 | Positive | 0 | 5426 | 0 | Negative |
| P17 | OCVM | UT | 611 | 3677 | **0.127** | NA | | |  |  |  |  |  |  | 0.127 | Positive | 0 | 6077 | 0 | Negative |
| P18 | OCVM | UT | NA | | | 38 | 338 | *0.1* |  |  |  |  |  |  | NA | NA | NA | | | NA |
| P19 | OCVM | UT | NA | | | 263 | 1407 | **0.151** |  |  |  |  |  |  | 0.151 | Positive | NA | | | NA |
| P20 | OCVM | UT | NA | | | 8 | 45 | *0.153* |  |  |  |  |  |  | NA | NA | 0 | 8705 | 0 | Negative |
| P21 | OCVM | TMU | 248 | 4447 | **0.046** | 171 | 1117 | 0.13 | 168 | 1033 | 0.137 | 179 | 1178 | 0.128 | 0.046 | Positive | NA | | | NA |
| P22 | OCVM | TMU | 525 | 5266 | **0.077** | 147 | 1050 | 0.12 | 99 | 770 | 0.112 |  |  |  | 0.077 | Positive | NA | | | NA |
| P23 | OCVM | TMU | NA | | | 9 | 427 | *0.21* | 10 | 33 | *0.231* |  |  |  | NA | NA | NA | | | NA |
| P24 | OCVM | TMU | NA | | | 54 | 372 | *0.126* | 7 | 32 | *0.18* |  |  |  | NA | NA | NA | | | NA |
| P25 | OCVM | TMU | NA | | | 484 | 3833 | **0.101** | 320 | 1986 | 0.131 |  |  |  | 0.101 | Positive | NA | | | NA |
| P26 | OCVM | TMU | NA | | | 58 | 388 | *0.129* | 16 | 148 | *0.099* |  |  |  | NA | NA | NA | | | NA |
| P27 | OCVM | TMU | NA | | | 196 | 1063 | 0.153 | 281 | 1569 | 0.146 | 282 | 1609 | **0.143** | 0.143 | Positive | NA | | | NA |
| P28 | OCVM | TMU | NA | | | 208 | 1297 | **0.134** | 69 | 439 | *0.134* |  |  |  | 0.134 | Positive | NA | | | NA |
| P29 | OCVM | TMU | NA | | | 267 | 1348 | **0.16** | 174 | 962 | 0.15 | 140 | 857 | 0.138 | 0.16 | Positive | NA | | | NA |
| P30 | OCVM | TMU | NA | | | 493 | 2585 | **0.149** | 156 | 750 | 0.169 |  |  |  | 0.149 | Positive | NA | | | NA |
| P31 | OCVM | TMU | NA | | | 180 | 1557 | **0.098** |  |  |  |  |  |  | 0.098 | Positive | NA | | | NA |
| P32 | OCVM | TMU | NA | | | 160 | 1093 | **0.124** | 140 | 757 | 0.129 |  |  |  | 0.124 | Positive | NA | | | NA |
| P33 | OCVM | TMU | NA | | | 205 | 1447 | 0.12 | 149 | 1348 | 0.097 | 506 | 3233 | **0.122** | 0.122 | Positive | NA | | | NA |
| P34 | OCVM | TMU | NA | | | 366 | 3189 | **0.093** | 300 | 1869 | 0.131 |  |  |  | 0.093 | Positive | NA | | | NA |
| P35 | OCVM | TMU | NA | | | 637 | 3151 | 0.157 | 1174 | 6275 | **0.153** |  |  |  | 0.153 | Positive | NA | | | NA |
| P36 | OCVM | TMU | NA | | | 307 | 1499 | **0.164** | 88 | 521 | 0.142 |  |  |  | 0.164 | Positive | NA | | | NA |
| P37 | OCVM | TMU | NA | | | 578 | 5648 | **0.077** | 374 | 4060 | 0.073 |  |  |  | 0.077 | Positive | NA | | | NA |
| P38 | OCVM | TMU | NA | | | 1672 | 7449 | **0.143** | 1155 | 4996 | 0.165 | 276 | 1569 | 0.145 | 0.143 | Positive | NA | | | NA |
| P39 | OCVM | TMU | 559 | 5769 | **0.075** | 460 | 4654 | 0.077 | 464 | 3179 | 0.117 | 203 | 1544 | 0.112 | 0.075 | Positive | NA | | | NA |
| P40 | OCVM | TMU | 0 | 5122 | **0** | 1 | 2422 | 0 | 0 | 2828 | 0 |  |  |  | 0 | Negative | NA | | | NA |
| P41 | OCVM | TMU | 541 | 4245 | **0.1** | NA | | |  |  |  |  |  |  | 0.1 | Positive | NA | | | NA |
| P42 | OCVM | TMU | 713 | 4657 | **0.118** | NA | | |  |  |  |  |  |  | 0.118 | Positive | NA | | | NA |
| P43 | OCVM | TMU | 681 | 5244 | **0.098** | NA | | |  |  |  |  |  |  | 0.098 | Positive | 0 | 9200 | 0 | Negative |
| P44 | OCVM | TMU | 1605 | 6621 | **0.154** | NA | | |  |  |  |  |  |  | 0.154 | Positive | 0 | 6269 | 0 | Negative |
| P1 | CCM | UT | 0 | 6577 | **0** | 0 | 1226 | 0 |  |  |  |  |  |  | 0 | Negative | 0 | 4903 | 0 | Negative |
| P2 | CCM | UT | 0 | 5154 | **0** | NA | | |  |  |  |  |  |  | 0 | Negative | 0 | 6520 | 0 | Negative |
| P3 | CCM | UT | 0 | 5190 | **0** | NA | | |  |  |  |  |  |  | 0 | Negative | 0 | 5615 | 0 | Negative |
| P4 | CCM | UT | 0 | 3594 | **0** | NA | | |  |  |  |  |  |  | 0 | Negative | 0 | 5486 | 0 | Negative |
| P5 | CCM | UT | 0 | 5251 | **0** | 0 | 8310 | 0 |  |  |  |  |  |  | 0 | Negative | 0 | 4720 | 0 | Negative |
| P6 | CCM | UT | 0 | 3417 | **0** | NA | | |  |  |  |  |  |  | 0 | Negative | 0 | 5806 | 0 | Negative |
| P7 | CCM | UT | 0 | 5382 | **0** | NA | | |  |  |  |  |  |  | 0 | Negative | 0 | 3994 | 0 | Negative |
| P8 | CCM | UT | 0 | 3630 | **0** | NA | | |  |  |  |  |  |  | 0 | Negative | 0 | 5692 | 0 | Negative |
| P9 | CCM | UT | 0 | 2948 | **0** | NA | | |  |  |  |  |  |  | 0 | Negative | 0 | 6337 | 0 | Negative |
| P10 | CCM | UT | 0 | 4011 | **0** | NA | | |  |  |  |  |  |  | 0 | Negative | 0 | 5373 | 0 | Negative |
| P11 | CCM | UT | 0 | 4373 | **0** | NA | | |  |  |  |  |  |  | 0 | Negative | 0 | 5959 | 0 | Negative |
| P12 | CCM | UT | 0 | 3593 | **0** | NA | | |  |  |  |  |  |  | 0 | Negative | 0 | 5303 | 0 | Negative |
| P45 | CCM | UT | NA | | | 0 | 4854 | **0** |  |  |  |  |  |  | 0 | Negative | NA | | | NA |
| P46 | CCM | UT | NA | | | 0 | 7446 | **0** |  |  |  |  |  |  | 0 | Negative | NA | | | NA |
| P13 | VH | UT | 0 | 5594 | **0** | NA | | |  |  |  |  |  |  | 0 | Negative | 0 | 4966 | 0 | Negative |
| P47 | dilated vein with organizing thrombus | TMU | 0 | 4193 | **0** | NA | | |  |  |  |  |  |  | 0 | Negative | 0 | 8811 | 0 | Negative |
| P48 | conjunctival capillary hemangioma | TMU | 0 | 5611 | **0** | NA | | |  |  |  |  |  |  | 0 | Negative | 0 | 11521 | 0 | Negative |

Italic font indicates results excluded due to the small number of WT droplets

Bold font indicates results selected as definitive MAFs of tissue samples

ddPCR; droplet digital polymerase chain reaction

OCVM, orbital cavernous venous malformation; CCM, cerebral cavernous malformation; VH, vertebral hemangioma

UT, The University of Tokyo; TMU, Tokyo medical university

FFPE, formalin-fixed paraffin-embedded

MUT, mutation; WT, wild-type; MAF, mutation allele frequency

NA, not available

**Table S3 ddPCR results for *GJA4* c.121G>T (p.Gly41Cys) of MACS-sorted cells from two prospectively collected OCVM samples**

| Participant ID | cell fraction | MUT droplets | WT droplets | MAF |
| --- | --- | --- | --- | --- |
| P43 | bulk tissue | 681 | 5244 | 0.098 |
|  | CD31-positive cells | 810 | 1475 | 0.342 |
|  | CD31-negative cells | 188 | 5545 | 0.02 |
| P44 | bulk tissue | 1605 | 6621 | 0.154 |
|  | CD31-positive cells | 567 | 903 | 0.383 |
|  | CD31-negative cells | 18 | 774 | 0.022 |

ddPCR; droplet digital polymerase chain reaction

MACS, magnetic-activated cell sorting

OCVM, orbital cavernous venous malformation

MUT, mutation; WT, wild-type; MAF, mutation allele frequency
